# Osteomalacia: a forgotten diagnosis of multiple bone fractures

**DOI:** 10.11604/pamj.2021.38.369.28987

**Published:** 2021-04-15

**Authors:** Karim Khezami, Mohamed Amine Bennour

**Affiliations:** 1 Faculty of Medicine of Tunis, University Tunis El Manar, Department of Orthopedic Surgery, Habib Bougatfa Hospital, Bizerte, Tunisia

**Keywords:** Osteomalacia, femoral neck, fracture

## Image in medicine

We present a case of a 53-year-old female patient with chronic anemia, with no other significant medical history and on no regular medications. The patient was brought to the accident and emergency department following found on the floor of her home after having tripped over a mat and henceforth being unable to mobilize and complaining of pain in her bilateral hip and her right leg. X-rays confirmed bilateral neck of femur fractures and fractures of the distal end of the tibia and fibula. The bones were extremely gracile and osteoporotic. Investigations demonstrated raised parathyroid hormone levels (199 pg/mL, normal range (nr) 15-72), with low vitamin D (7 ng/mL, nr 50-100) and adjusted calcium (1.87 mmol/L, nr 2.2-2.6) levels. We have eliminated the diagnosis of myeloma. Calcium and vitamin D were replaced prior to the insertion of bilateral femoral nails.

**Figure 1:**
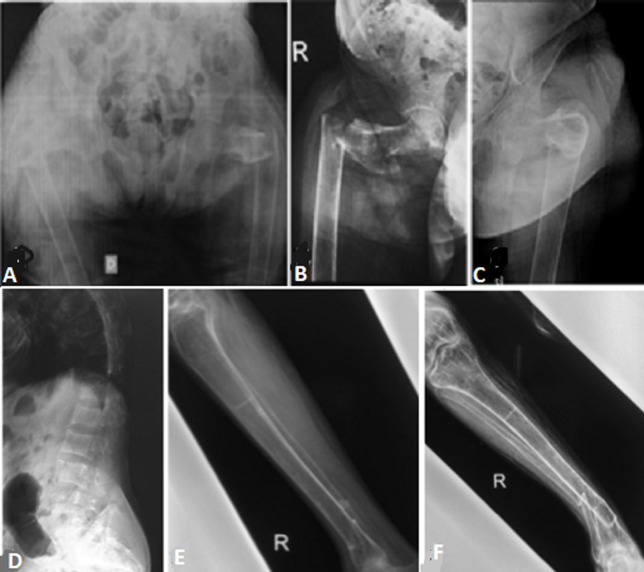
A) bilateral proximal femoral fractures associated with low energy; B) right subtrochanteric femoral fracture; C) left subtrochanteric femoral fracture; D) diffuse demineralization of the spine: osteoporotic-like pattern; (E,F) supramalleolar fracture of tibia and fibula

